# A simple algebraic cancer equation: calculating how cancers may arise with normal mutation rates

**DOI:** 10.1186/1471-2407-10-3

**Published:** 2010-01-05

**Authors:** Peter Calabrese, Darryl Shibata

**Affiliations:** 1 Program in Molecular and Computational Biology, Department of Biological Sciences, University of Southern California, Los Angeles, CA 90089, USA; 2 Departments of Pathology, University of Southern California Keck School of Medicine, Los Angeles, CA 90033, USA

## Abstract

**Background:**

The purpose of this article is to present a relatively easy to understand cancer model where transformation occurs when the first cell, among many at risk within a colon, accumulates a set of driver mutations. The analysis of this model yields a simple algebraic equation, which takes as inputs the number of stem cells, mutation and division rates, and the number of driver mutations, and makes predictions about cancer epidemiology.

**Methods:**

The equation [*p *= 1 - (1 - (1 - (1 - *u*)^*d*^)^*k*^)^*Nm *^] calculates the probability of cancer (*p*) and contains five parameters: the number of divisions (*d*), the number of stem cells (*N *× *m*), the number of critical rate-limiting pathway driver mutations (*k*), and the mutation rate (*u*). In this model progression to cancer "starts" at conception and mutations accumulate with cell division. Transformation occurs when a critical number of rate-limiting pathway mutations first accumulates within a single stem cell.

**Results:**

When applied to several colorectal cancer data sets, parameter values consistent with crypt stem cell biology and normal mutation rates were able to match the increase in cancer with aging, and the mutation frequencies found in cancer genomes. The equation can help explain how cancer risks may vary with age, height, germline mutations, and aspirin use. APC mutations may shorten pathways to cancer by effectively increasing the numbers of stem cells at risk.

**Conclusions:**

The equation illustrates that age-related increases in cancer frequencies may result from relatively normal division and mutation rates. Although this equation does not encompass all of the known complexity of cancer, it may be useful, especially in a teaching setting, to help illustrate relationships between small and large cancer features.

## Background

The motivation for this article is to present an easy to understand equation that illustrates how cancers can arise within a lifetime from relatively normal mutation and division rates. Given the multiplicity and greater sophistication of many other cancer models, it is primarily presented as a teaching tool to demonstrate how cancers may result as mutations accumulate in stem cells under a very simple scenario. The goal is to illustrate to a wider audience (an average college graduate) that many numerical aspects of cancer biology may be described mathematically. (A short slide presentation summarizing the major points is provided as Additional file 1.)

A more formal analysis of this equation was previously published [1], which predicted human colorectal cancers could arise with relatively normal mutation and division rates, and the current presentation is a simpler, algebraic version that may be easier to understand and manipulate. As recently noted [2], algebraic methods are often more intuitive and easy to understand than differential equations. Since its publication, the mutational landscape of colorectal cancer genomes has been better characterized [3-5]. An interesting observation is that the mutation frequency in a cancer genome is less than one mutation per 100,000 bases, which is consistent with relatively normal mutation and division rates [3]. This new experimental data motivates us to revisit how cancers may arise with normal division and mutation rates. Because all cells initially have normal mutation and division rates, it is possible to estimate the relative roles of old age and "bad luck" (i.e. a parsimonious pathway because functional changes are unnecessary during progression), versus a necessity of overcoming specific anti-cancer barriers during progression to cancer.

Cancer results from the accumulation of multiple alterations in a single transformed cell [6]. Even if the probability of transformation is extremely low for a single cell, cancer could arise by chance within a lifetime if many cells are at risk. The number of cells at risk and the number cells that transform can be inferred from cancer epidemiology. In America, millions of individuals are at risk and every year thousands of cancers are diagnosed. Many common cancers exhibit an increase in incidence with age, which can be described by a simple equation [7].(1)

Parameters are *p *(probability of cancer), *b *(a constant), *t *(age of individual), and *k *(the number of rate-limiting stages). The equation fits the epidemiology of colorectal cancer when *k *is 5 or 6.

This equation does not include many biological parameters, which are presumably incorporated into its constant "*b*". Intuitively, cancer incidence should increase with greater numbers of cells at risk, with greater numbers of cell divisions, and with higher mutation rates. Here we present a simple algebraic equation that relates small biological features (adult stem cells and their niches, tissue size, numbers of rate-limiting driver mutations, and mutation rates) with the epidemiology of colorectal cancer.

## Methods

Normal mutation rates are low and around one mutation per billion bases per division [8], which extrapolates to a probability of mutating a single specific gene of ~1,000 base pairs in a single division as 10^-6^. The probability of cancer (*p*) after a single division is extremely low if six rate-limiting (*k*) mutations are required.(2)

The probability of cancer is 10^-36 ^when the mutation rate (*u*) is 10^-6 ^mutations per gene per division and *k *is six. It is highly improbable that cancer will arise in a single cell after a single division. A more useful calculation is the probability of cancer after the many divisions that occur during a human lifetime, and in just one of the many cells at risk in the body. The approach is based on the trick that the probability of "something" plus the probability of "not something" equals one. The probability of not accumulating a critical mutation (1-*u*) in one cell lineage after a certain number of divisions (*d*) is:(3)

With more divisions, the probability of no mutation decreases. It follows that the probability of mutation after *d *divisions is:(4)

For multiple (*k*) genes:(5)

The above equation calculates the probability of a single cell accumulating all *k *driver mutations after *d *divisions. It follows that the probability of not accumulating all *k *mutations in a single cell after *d *divisions is:(6)

**Figure 1:**
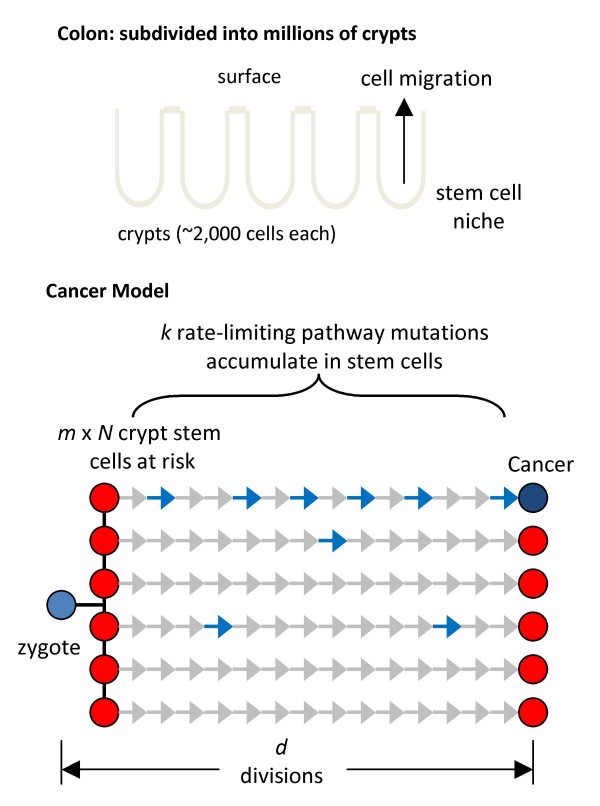
**Progression model**. A colon consists of ~15 million epithelial clonal units called crypts. Each crypt contains ~2,000 cells and is maintained by a smaller number of stem cells that are present near its base. A cancer genealogy starts at the zygote and ends when the present day cancer cell is detected. The predominant phenotype in this genealogy is a stem cell phenotype because visible tumors are rare before the age of 50 years. Many colorectal crypt stem cells (*m *× *N*) are produced early in life, and these lineages in different crypts independently divide and accumulate passenger (gray arrows) and driver (blue arrows) somatic mutations with aging. Transformation occurs by chance when an appropriate combination of *k *rate-limiting driver pathway mutations first accumulates in a single stem cell lineage.

The probability that a single cell accumulates six driver mutations is low. However, cancer arises when the first cell out of many at risk within an individual transforms, which is considerably earlier than the average cell. For an organ the probability of cancer depends on the number of cells at risk, which is fewer than the total number of cells because mutations can only accumulate in long-lived stem cell lineages. For the colon (Fig 1), the number of cells at risk is the number of stem cells per crypt (*N*) multiplied by the total number of clonal units or crypts (*m*).(7)

It follows that the probability of cancer (*p*) for a single individual is 1 minus the above equation.(8)

Equation [8] is an algebraic representation of an analysis published earlier [1] for a probabilistic model of colorectal cancer that starts from birth and ends when the first stem cell (out of many at risk) accumulates a critical number of *k *rate-limiting driver mutations (Fig 1). The model assumes all mutations (drivers and passengers) are initially selectively neutral and arise as replication errors. This algebraic format may be easier to understand, especially in a teaching setting, and can be manipulated with an Excel spreadsheet (SOM). There are five parameters and several assumptions, which allow considerable freedom to "curve fit" almost any data (Tables 1 and 2, Fig 1). However, parameter values are constrained by the known biology of normal human tissues and their cancers. To illustrate its potential utility, Equation [8] is applied to various data sets.

**Table 1:** Model Parameters

Parameter	Description	Colorectal Cancer With Specific Gene Targets*	Colorectal Cancer With Pathway Gene Targets**
*k*	rate-limiting stages	5 driver gene mutations	6 pathway mutations

*m*	number of crypts	15,000,000	15,000,000

*n*	stem cells per crypt	40	8

*u*	target mutation rate	1 × 10^-6 ^per gene per division	3 × 10^-6 ^per pathway per division

*d*	divisions since birth	once every four days	once every four days

*p*	probability of cancer	-	-

*In this model, five specific driver genes must be mutated for transformation

**In this model, six driver genes must be mutated for transformation, but because multiple genes in the same pathway are functionally equivalent, the mutational target or numbers of base pairs at risk are larger than when mutations must occur in specific driver genes.

**Table 2:** Model Assumptions For An Average Repair Proficient Colorectal Cancer

Parameter	Assumptions	Comments
driver mutations or rate-limiting stages (*k*)	the same for all cancers, transformation or growth does not occur until all driver mutations accumulate	value is unknown and is inferred to fit the epidemiology, growth is likely to precede transformation but most mutations likely accumulate in normal stem cells

number of crypts (*m*)	does not change during life	crypt number may vary between individuals

stem cells per crypt (*n*)	does not change during life	value is unknown (minimum of one per crypt), inferred to fit the epidemiology.

mutation rate (*u*)	does not change during life	value may differ between genes but is ~10^-10 ^to 10^-9 ^per base per division

stem cell divisions since birth (*d*)	constant division rate during life	value is unknown but may be as high as once per day [13]

probability of cancer (*p*)	no significant time between transformation and diagnosis	data from cancer epidemiology, lag time may vary between patients

## Results

### Epidemiology of colorectal cancer

**Figure 2:**
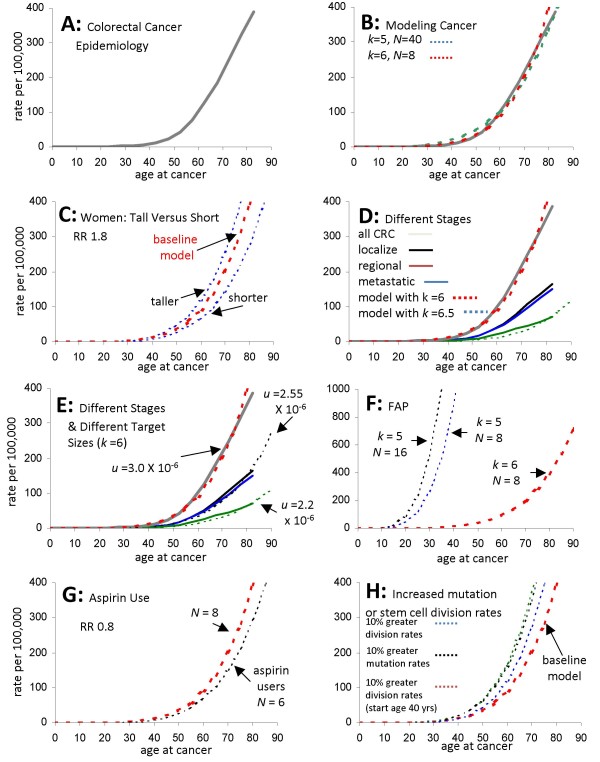
**Colorectal cancer**. **A**: Epidemiology documents increased colorectal cancer incidence with age (data from Ref [9]). **B**: Equation [8] can approximate epidemiology (gray line) with 5 *k *rate-limiting mutations and 40 niche stem cells (green dotted line), or 6 *k *rate-limiting pathway mutations and 8 niche stem cells (red dotted line). See Table 1 for other parameters. **C**: Increased cancer risks with height in women can be modeled by changing colon lengths or crypts per colon (*m*). A 1.8 relative risk between the shortest and tallest quintiles can be modeled by changing lengths 28.6% (blue dotted lines) relative to the average colon (red dotted line). Crypts in the shortest quintile are 80% of the tallest quintile. **D**: Metastatic cancer has a lower incidence and arises later than localized or regional cancer, which can be modeled (dotted lines) by increasing *k *rate-limiting pathway mutations from 6 to 6.5. **E**: Lower cancer subtype incidence can also be modeled (dotted lines) with *k *= 6 and smaller mutational target sizes (*u*). For all cancers, *u *= 3 × 10^-6 ^but is 2.55 × 10^-6 ^for localized or regional cancers, and 2.2 × 10^-6 ^for metastatic cancers (also see Fig 4). **F**: Earlier FAP cancer incidence can be modeled by decreasing *k *from six in sporadic cancers to five in FAP. This incidence shifts to even younger ages by also increasing crypt niche stem cells (*N*) from 8 to 16. **G**: A cancer relative risk of 0.8 with chronic aspirin use can be modeled by decreasing crypt niche stem cells from 8 to 6. **H**: A similar increase in cancer occurs with a 10% increase in stem cell division rate (green dotted line), or a 10% mutation rate increase (black dotted line). If division increases later in life, the cancer incidence increase is lower (blue dotted line, 10% increase in stem cell division rate starts at 40 years of age).

The incidence of colorectal cancer increases with age (Fig 2A). The age-incidence data for colorectal cancer were obtained from the Surveillance, Epidemiology, and End Results Program (SEER 11 Regs Public-Use, Nov 2001 Sub (1992-1999)), a population-based registry in the United States of America that records all cancers regardless of clinical treatment [9]. A total of 108,275 records were analyzed for ages at cancer selected by site (colon and rectum), race (white), histology (adenocarcinoma, ICD-0-2 codes 8000-8500), and stage (localized, regional, or distant). Equation [8] calculates a cumulative probability of cancer after *d *divisions, which is converted to incidence by segregating new cancers into five-year intervals as with the SEER data.

This epidemiology can be reconstructed with Equation [8] and parameter values consistent with colon biology (Table 1). The number of crypts per colon is ~15 million [10]. The mutation rate is set at 10^-6 ^per division per gene [8]. The division rate is set at one division every four days, as modeled in a recent analysis [8]. Uncertain are the numbers of stem cells per crypt and the number of rate-limiting stages or mutations.

Curve fitting with five *k *rate-limiting mutations and 40 stem cells per crypt approximates the epidemiologic data (Fig 2B). However, recent cancer genome data suggest that functional or regulatory pathways rather than specific sets of genes are more relevant oncogenic targets because driver mutations are diverse [4,5,11,12]. Mutation of several genes in a regulatory pathway may be functionally equivalent. If three genes are at risk in a pathway, then the probability of mutation (*u*) of any one of the three genes in a single division is 3 × 10^-6 ^instead of 1 × 10^-6 ^with a single gene target (i.e. the mutation target is 3,000 bases instead of 1,000 bases). Curve fitting with six *k *rate-limiting pathway mutations and eight stem cells per crypt also approximates the epidemiologic data (Fig 2B and Table 1). Equation [8] with six *k *rate-limiting driver pathway mutations will be subsequently used for further analysis because of a better conceptual fit with the idea that regulatory pathways rather than specific single genes are altered in cancer [5].

Numbers of divisions between the zygote and a cancer genome can be estimated by measuring total numbers of somatic driver and passenger mutations.(9)

**Figure 3:**
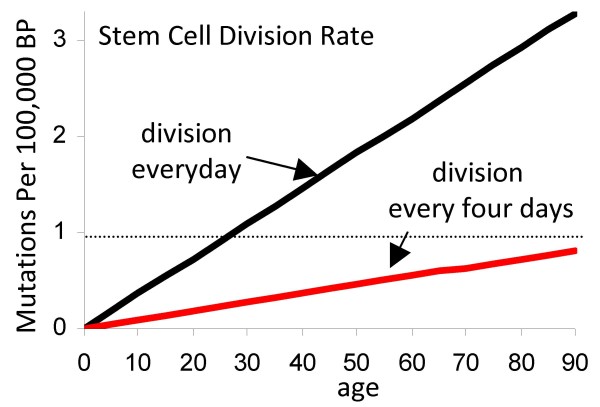
**Division rates can be inferred from cancer genome mutation frequencies and Equation [9]**. With a rate of 10^-9 ^mutations per base per division, mutations accumulate with aging in mitotic tissues. Cancer genomes generally have less than one somatic mutation per 100,000 bases (dotted line). A division rate of once every four days rather than once a day is more consistent with observed cancer genome mutation frequencies. In our model, most cancer somatic mutations accumulate earlier in life in normal appearing stem cells (Fig 1).

Clonal somatic mutation frequencies in most repair proficient colorectal cancers are less than one mutation per 100,000 bases [3,4]. With a mutation rate of 10^-9 ^per base per division (*U*), a cell division rate of once per day would yield ~2.6 mutations per 100,000 bases whereas a division rate of once every four days [8] would yield ~0.65 mutations per 100,000 bases at an age of 70 years (Fig 3).

Most of the divisions to cancer likely occur in stem cells because the genealogy of a cancer cell starts at the zygote and ends at the present day cancer genome (Fig 1). Phenotype varies along this genealogy, but a crypt stem cell phenotype occupies the longest interval because visible tumorigenesis before the age of 50 years is rare. The stem cell division rate is uncertain because human crypt stem cells have not been conclusively identified or characterized. In mice, crypt stem cell division rates were estimated at once per day, using a new potential stem cell marker Lgr5 [13]. A human stem cell division rate of once every four days and the parameter values in Table 1 approximate the epidemiology and the observed mutation frequencies of colorectal cancers.

### Measuring colon lengths from cancer incidence

To further test the utility of Equation [8], we apply it to another data set. The equation predicts the incidence of colorectal cancer will increase with the number of crypts. Colon lengths are difficult to measure because the organ is elastic, but taller individuals generally have longer colons [14]. Taller individuals also appear to have higher risks for colon cancer. In one study, the relative risk of cancer increased 1.4 in men and 1.8 in women between the tallest and shortest quintiles of individuals [15].

One can model these cancer frequency changes with about 16.7% fewer crypts in the shorter quintile and 16.7% more crypts in the taller quintile for men, and 28.6% fewer and 28.6% more crypts in women (Fig 2C). Colon lengths may vary over 2-fold [16], allowing for the variation predicted with the equation. This example indicates how a small biological feature (*m *or the number of crypts) is interrelated with cancer risks and can be indirectly measured from cancer epidemiology.

### Estimating numbers of mutations required for metastases

**Figure 4:**
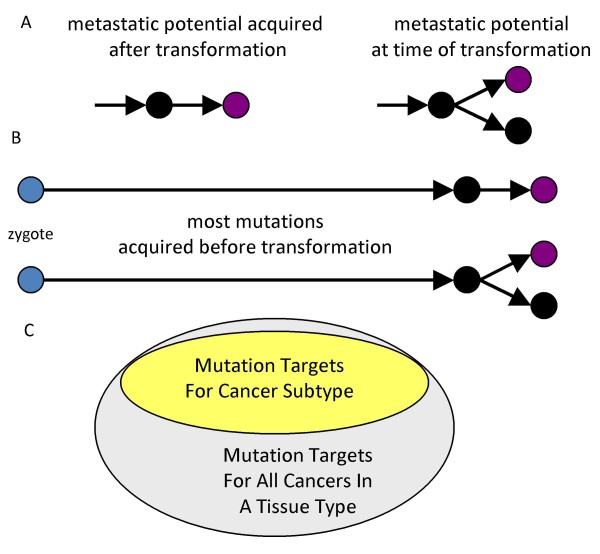
**Metastatic cancer**. **A**: The ability to metastasize may require additional mutations after transformation or be present at the time of transformation **B**: Diagramming the entire genealogy of a cancer reduces the illusion that a metastases should be significantly different from its primary tumor because mutations accumulate throughout life and it is unlikely that a cancer will be asymptomatic for many years. **C**: Subclassification reduces the relative incidence of each cancer subtype. Equation [8] may still apply to cancer subtypes without changing the numbers of *k *rate-limiting pathway mutations by subdividing the mutational target sizes for each subtype. The genes that can be mutated and lead to metastatic cancer may be a smaller subset of the genes that can be mutated and lead to any type of colorectal cancer.

Metastases may require additional alterations after transformation (Fig 4) that allow tumor cells to invade, migrate, and colonize distant sites [17]. Alternatively, many cancers may already have the ability to metastasize at the time of transformation [18,19]. Metastatic colorectal cancer arises somewhat later in life compared to localized or regional cancer (Fig 2D, data from Ref [9]). The later and lower incidence of metastatic cancer can be modeled with Equation [8] and the same parameters as for all colorectal cancer except *k *is increased from 6 to 6.5 (Fig 2D).

The biological meaning of "half" a rate-limiting pathway mutation is unclear, and may indicate that Equation [8] does not readily apply after the onset of visible tumorigenesis (see below). Alternatively, a parameter change that can decrease the incidence of a cancer subtype without changing *k *is a decrease in the size of the mutational pathway targets, which effectively lowers the mutation rate *u*. Progression to a particular cancer subtype may require a smaller subset of all possible mutational targets for a general type of cancer (Fig 4). Whereas *u *is 3 × 10^-6 ^for all colorectal cancers, localized or regional cancers, and metastatic cancers appear to have smaller mutational targets, respectively 2.55 × 10^-6 ^and 2.2 × 10^-6 ^(Fig 2F). Instead of linear progression (Fig 4A), this modeling implies that metastatic cancers also require only six rate-limiting driver pathway mutations that confer both transformation and the ability to metastasize.

Whether or not the capability for metastasis is present at transformation or acquired after transformation, the geometry of Equation [8] predicts minimal differences in numbers of mutations between a primary and its metastases because the interval before transformation is typically much greater than the interval after transformation (Fig 4B). For example, if transformation occurred at 78 years of age and a metastatic cancer is removed two years later, only 2.5% of the cancer genealogy interval accumulated after transformation. A recent study also found few mutational differences between a metastatic tumor and its primary [8]. On average 97% of the mutations found in the metastatic lesion were also detected in its primary.

### Familial cancers and germline mutations (*k-1*)

Familial cancers are characterized by cancer at earlier ages and germline inactivation of one allele of an important tumor suppressor gene. For example, familial adenomatous polyposis (FAP) is characterized by heterozygous germline APC mutations [20], and APC somatic mutations are present in most sporadic colorectal cancers [5]. Decreasing the number of rate-limiting pathway mutations from six (sporadic cancer value) to five recreates the earlier age onset of cancer in FAP (Fig 2F).

### Effective numbers of crypt stem cells at risk for transformation

The number of crypt stem cells is difficult to measure directly because of the lack of specific or sensitive markers. Estimates of stem cell numbers per crypt range from one to forty in mice [21]. Human crypt stem cell numbers are more uncertain as experimental manipulations are limited.

**Figure 5:**
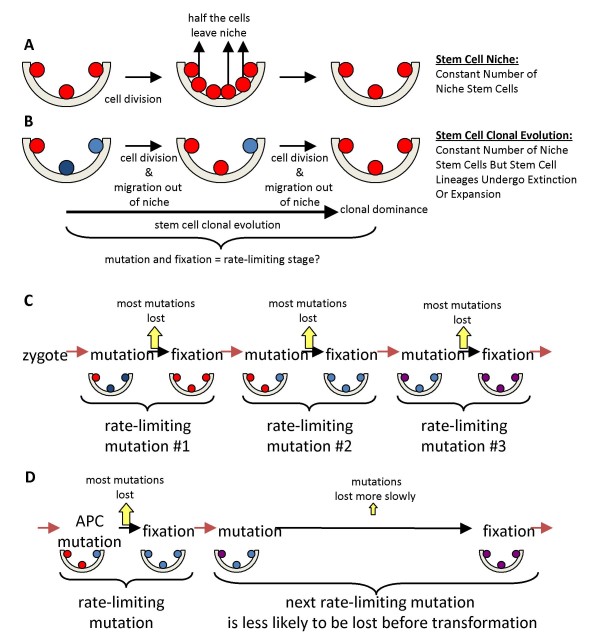
**Niche stem cell clonal evolution**. **A**: A hypothetical niche with three stem cells. In niches, stem cell numbers rather than lineages are constant. With stem cell division, half the daughter cells leave the niche and differentiate. **B**: Stem cell lineages are distinguished by different colors. Niche stem cells are maintained by a population mechanism rather than strictly asymmetric divisions. Sometimes both daughter cells leave the niche (lineage extinction), balanced by two daughter cells that remain within the niche (lineage expansion). Eventually clonal evolution occurs when all but one present day stem cell lineage becomes extinct, which appears to recur about every eight years in human crypts [10]. **C**: Crypt stem cell clonal evolution complicates mutation accumulation because most mutations are lost when their stem cells become extinct. Early mutations can only accumulate or become fixed if they occur in the stem cell that attains clonal dominance. A driver change acquired early in life therefore requires both mutation and fixation, which may help explain why the increased cancer incidence with aging appears to occur through multiple distinct rate-limiting stages [7]. **D**: Crypt stem cell clonal evolution appears to take longer after an APC mutation, which decreases the likelihood that the next driver mutation will be lost before transformation. Certain APC mutations may shorten pathways to cancer because they are present in nearly all colorectal cancer genomes.

A complication of stem cell numerical estimates is that mammalian stem cells appear to be maintained by niches [22]. In niches, stem cell numbers but not lineages are constant, because turnover involves a population mechanism (Fig 5). A stem cell usually divides asymmetrically to produce one stem and one differentiated daughter, but sometimes a stem cell will produce two differentiated daughter (lineage extinction) balanced by another stem cell that produces two stem cell daughters (lineage expansion). Eventually all but one present-day stem cell lineage becomes extinct, which is equivalent to the clonal evolution of tumor progression [6] except there are no changes in visible phenotype or population size. Stem cell clonal evolution appears to recur about every eight years in human colon crypt niches [10]. As previously discussed [1], the effective number of stem cells at risk for cancer (*N*) depends on how often stem cell clonal evolution recurs and is between one and the total number of niche stem cells. Less frequent clonal evolution may increase cancer risks because stem cell lineages persist longer and the effective number of stem cells at risk for progression is greater.

Niches modify mutation accumulation. Most early mutations are lost because most stem cell lineages become extinct during crypt clonal evolution. The niche serves as a crucible---early mutations in a cancer genealogy must also achieve fixation by occurring in the single stem cell that attains crypt clonal dominance. Because the niche population size is small, neutral or even mutations that confer a slight disadvantage may become fixed by chance or drift [23] rather than selection within a crypt. A requirement for both mutation and subsequent fixation (Fig 5C), or two hurdles with each rate-limiting stage (or "relatively rare event" [7]) may help make cancer even rarer [1].

### Contingency and WNT-signaling

Transformation of a stem cell lineage later in life is contingent on its persistence earlier in life despite periodic threats of extinction during niche clonal evolution, which may help explain why APC mutations are found in nearly all colorectal cancers [5]. Crypt stem cell survival depends on several signaling pathways. WNT signaling appears necessary for crypt stem cell survival, and APC is a central regulator of the Wnt pathway [24,25].

FAP individuals are born with normal appearing colon crypts but have heterozygous APC germline mutations. Certain APC mutations confer dominant-negative effects with up-regulation of Tcf-B-catenin-mediated transcription in experimental systems [26]. Some heterozygous APC mutations appear to decrease cell mobility [27], which may enhance survival of its stem cell relative to surrounding wild type stem cells that more readily migrate out of the niche. In this way, certain APC mutations may be more common in colorectal cancers because when acquired earlier in life, they also favor persistence of its stem cell through subsequent crypt clonal evolution cycles. Simplistically, APC mutations may favor progression with a minimum of divisions because fixation of subsequent driver mutations is less imperative (Fig 5D). Interestingly, APC may undergo sequential mutation and selection during progression [28].

Passenger methylation patterns in normal appearing FAP crypts are more diverse than non-FAP crypts, consistent with enhanced stem cell survival [29]. This enhanced stem cell survival effectively doubles the number of FAP niche stem cells and increases the average crypt stem cell clonal evolution interval from eight to 30 years [29]. The doubling of FAP crypt stem cells increases the risk of cancer (Fig 2F), and this addition effect of certain APC mutations along with one fewer rate-limiting *k *mutations better fits the observed incidence of FAP cancer with aging [28].

Conversely, inhibition of the Wnt-signaling pathway may effectively decrease niche stem cell numbers and reduce cancer. Non-steroidal anti-inflammatory drugs inhibit Wnt-signaling and down regulate Tcf-B-catenin transcription [30,31]. Aspirin use is associated with reduced colorectal cancer, with relative risks of about 0.8 compared to non-aspirin users [32]. A 25% reduction in effective stem cell number (*N*) from 8 to 6 per crypt can account for the ~0.8 relative risk decrease with aspirin use (Fig 2G).

### Mutation rates versus cell division

More mutations will accumulate with increased mutation or cell division rates [33]. Inflammatory bowel disease (IBD) is associated with increased cancer risks, which increases with the length and extent of disease [34]. IBD was modeled with Equation [8] with either a 10% increase in mutation or stem cell division rates (Fig 2H). The predicted effect is a ~1.8-fold increased relative risk of cancer. Stem cell proliferation or mutation rate changes appear to be equivalent with respect to cancer risks.

### A mutation deficit: a legacy from normal colon

**Figure 6:**
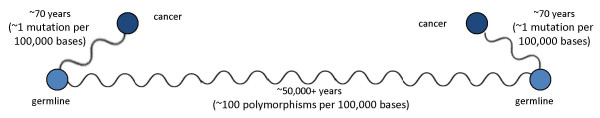
**Differences among normal genomes are ~100-fold greater that the differences between cancers and their germline genomes, perhaps reflecting the relative times or divisions since a common ancestor**.

Cancer genome projects provide numbers to compare the relative amounts of "genomic instability" thought to riddle cancers. The difference between a cancer genome and its germline sequence is less than one base per 100,000 [3], which is ~100-fold less than the variation (~one base per 1,000) between normal germline human genomes. Much greater sequence differences are present among individuals than between a cancer and its germline genome---the first cell that transforms requires relatively few mutations to "find" an appropriate combination of driver mutations (Fig 6). A "normal" human genome can absorb many more changes than found in a typical repair proficient cancer. The diverse mutation combinations between cancer genomes [5] may reflect the much larger variation between their starting germline genomes.

Potentially there are many more mutations secondary to copy number changes from chromosomal instability or CIN [35]. However, early sequencing studies suggest that relatively few DNA breaks may underlie CIN. For example, less than one hundred somatically acquired breakpoint sequences per lung cancer cell line (~1 breakpoint per 10,000,000 bases) were detected with genome-wide massively parallel paired-end sequencing [36].

Logically the first cell that transforms requires fewer divisions than subsequent cells. Alternative but longer, less parsimonious pathways may not be observed simply because transformation cannot occur within a lifetime. A start from conception and decades in normal colon may also help explain why the numbers of divisions to cancer appear consistent with near normal division and mutation rates [3], because uncontrolled proliferation may be limited to the relatively short terminal neoplastic phase of a cancer genealogy. Decades in normal colon can also help explain why pathways to cancer almost always collect an APC mutation that may favor persistence during niche clonal evolution and lessen a fixation requirement for subsequent driver mutations.

## Discussion

Cancer modeling has a long history (see for example ref [37]) and it is possible to fit many models to cancer data. Such modeling is complicated because many parameter values are uncertain and likely to differ between individuals, populations, and through time. Ideally, experimentalists and modelers interact, but many cancer equations are incomprehensible to many students and experimentalists. The current equation incorporates some of the assumptions in other cancer models (see Table 2), but its algebraic format may be easier to understand and manipulate [2].

What "causes" cancer? This model examines whether colorectal cancers can arise within a lifetime from normal division and mutation rates, and without serial selection and clonal expansion (a parsimonious pathway). Whereas the accumulation of sufficient numbers of driver mutations might be highly unlikely with normal mutation rates [38], new experimental data illustrate that colorectal cancer mutation frequencies are relatively low and consistent with normal mutation and division rates [3]. This new data constrains models because proliferation or mutation rates do not have to be and are not significantly altered during most of progression. Stem cells, which are the long-lived lineages that can accumulate mutations during progression [39], might seldom divide, but recent studies in mice suggests crypt stem cells are not quiescent but actively divide about once per day [13]. An important distinction is that an individual gets cancer when the first cell and not the average cell accumulates a critical number of driver mutations.

Here we illustrate that mutation accumulation from normal cell replication can account for the low per cell transformation rates and low cancer genome mutation frequencies. Progression to cancer is complex and variable, but certain biological features are likely to be fundamentally important when averaged over many individuals and many years. These factors are the number of divisions (*d*), the number of stem cells (*N *× *m*), the number of critical rate-limiting driver pathway mutations (*k*), and the mutation rate (*u*). The probability that at least one stem cell accumulates the required number of driver mutations in an individual's lifetime is substantially greater than the probability a typical stem cell acquires these mutations. Given a 5% risk of colorectal cancer by 100 years of age, only five cells in 100 individuals transform after 100 years. There are ~15 million crypts per colon and therefore at least ~15 million stem cells at risk for colorectal cancer in an individual. Therefore, only ~five of 1.5 billion crypt stem cell lineages transform within a 100 years, or a single transformation event per ~30 billion crypt stem cell years (stem cell lineage transformation efficiency ~3 × 10^-9^). Chance and the enormous variation generated by replication errors in millions of stem cell lineages may be sufficient for the selection of low frequency cancer phenotypes within a lifetime.

A probabilistic description of cancer has several aspects consistent with cancer genome data, which show relatively low mutation frequencies, diverse combinations of mutations between different tumors, and a high proportion (>80%) of neutral passenger mutations [4,5,11,12]. Equation [8] models random mutation that starts from conception and therefore the numbers and types of mutations in a cancer genome is highly dependent on what happens in normal colon (Fig 1). Mutations (predominantly passenger mutations) may arise as replication errors, and cancer results by chance from rare and diverse driver mutation combinations that confer a malignant phenotype in a single cell. Certain APC mutations may be common in colorectal cancer because they enhance stem cell survival during niche clonal evolution and shorten pathways by effectively increasing subsequent numbers of stem cells at risk. The similar base mutation spectrum in colorectal, pancreatic, and glial tumors [11] is consistent with a common underlying mechanism such as replication errors.

A cancer model that includes epidemiology data needs an age parameter. Implicit in Equation [8] is that progression starts at conception and most mutations accumulate in normal appearing colon (Fig 1). Once visible tumorigenesis occurs, this equation does not readily apply because it calculates the risk of the entire colon and does not model the adenoma-cancer sequence [20]. However, progression to cancer may be dominated by its passage in normal colon because tumors before the age of 50 years are rare. The accumulation of somatic driver mutations in normal tissues is poorly documented, but mouse models demonstrate that many oncogenic mutations are also compatible with normal phenotypes [40], illustrating that some driver mutations can potentially arise earlier in life and persist in normal colon. Transformation of primary human cells has been engineered in vitro, but tumorigenesis in nude mice required the simultaneous combination of all three changes in a single cell [41].

This simple equation does not include copy number or epigenetic variations, or the very likely possibility that error and division rates may change during progression, and should be viewed as an exploratory or teaching tool. Many other quantitative models of cancer have been published, include a model of cancer genome data [42], but the algebraic format of this equation may be more familiar to students, which can also be manipulated with an Excel spreadsheet (see Additional file 2). By this equation, cancer is "caused" by replication errors, a large number of cells at risk, and "bad luck" [43], with cancer risks increased by stem cell divisions that normally occur with aging [33]. The examples analyzed with Equation [8] illustrate that subtle rather than dramatic cell changes are consistent with risk changes measured in large populations. From a broader perspective, its "integrative" nature relates how cancer incidence may depend on effective stem cell numbers, division and error rates, and numbers of required rate-limiting driver pathway mutations. Many progression pathways from the zygote are possible, but the shorter, parsimonious ways may allow cancers to appear within a lifetime.

## Conclusions

The equation *p *= 1 - (1 - (1 - (1 - *u*)^*d*^)^*k*^)^*Nm *^illustrates that age-related increases in cancer frequencies may result from relatively normal division and mutation rates. Although this equation does not encompass all of the known complexity of cancer, it may be useful, especially in a teaching setting, to help illustrate relationships between small and large cancer features.

## Competing interests

The authors declare that they have no competing interests.

## Authors' contributions

PC developed the equations and helped to draft the manuscript. DS translated the equations to cancer biology and helped to draft the manuscript. All authors read and approved the final manuscript.

## Pre-publication history

The pre-publication history for this paper can be accessed here:

http://www.biomedcentral.com/1471-2407/10/3/prepub

## Supplementary Material

Additional file 1 **Short slide presentation of the major points of the equation**. Powerpoint slides (N = 5) Click here for file

Additional file 2 **Spreadsheet for calculations with the equation**. Excel spreadsheet Click here for file
