# Supplementary material for: A simple algebraic cancer equation: calculating how cancers may arise with normal mutation rates
Source: BMC Cancer. 2010 Jan 5;10:3. doi: 10.1186/1471-2407-10-3 (PMC2829925; doi:10.1186/1471-2407-10-3)
Supplement: Additional file 1 — Short slide presentation of the major points of the equation. Powerpoint slides (N = 5) [file 1471-2407-10-3-S1.PPT]

## Slide 1
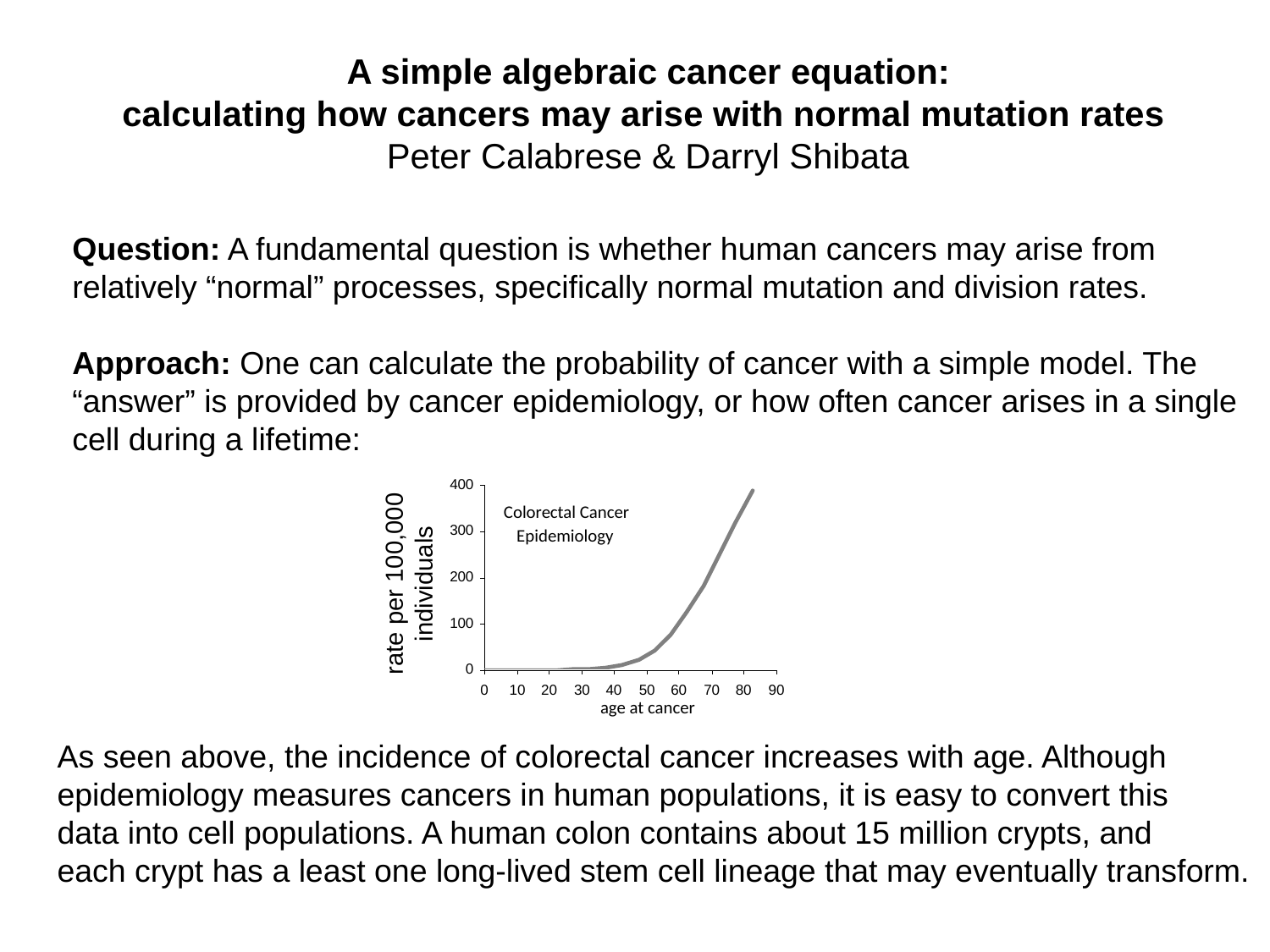

A simple algebraic cancer equation:
calculating how cancers may arise with normal mutation rates
Peter Calabrese & Darryl Shibata
Question: A fundamental question is whether human cancers may arise from
relatively “normal” processes, specifically normal mutation and division rates.
Approach: One can calculate the probability of cancer with a simple model. The
“answer” is provided by cancer epidemiology, or how often cancer arises in a single
cell during a lifetime:
 Colorectal Cancer
 Epidemiology
rate per 100,000
individuals
age at cancer
As seen above, the incidence of colorectal cancer increases with age. Although
epidemiology measures cancers in human populations, it is easy to convert this
data into cell populations. A human colon contains about 15 million crypts, and
each crypt has a least one long-lived stem cell lineage that may eventually transform.

## Slide 2
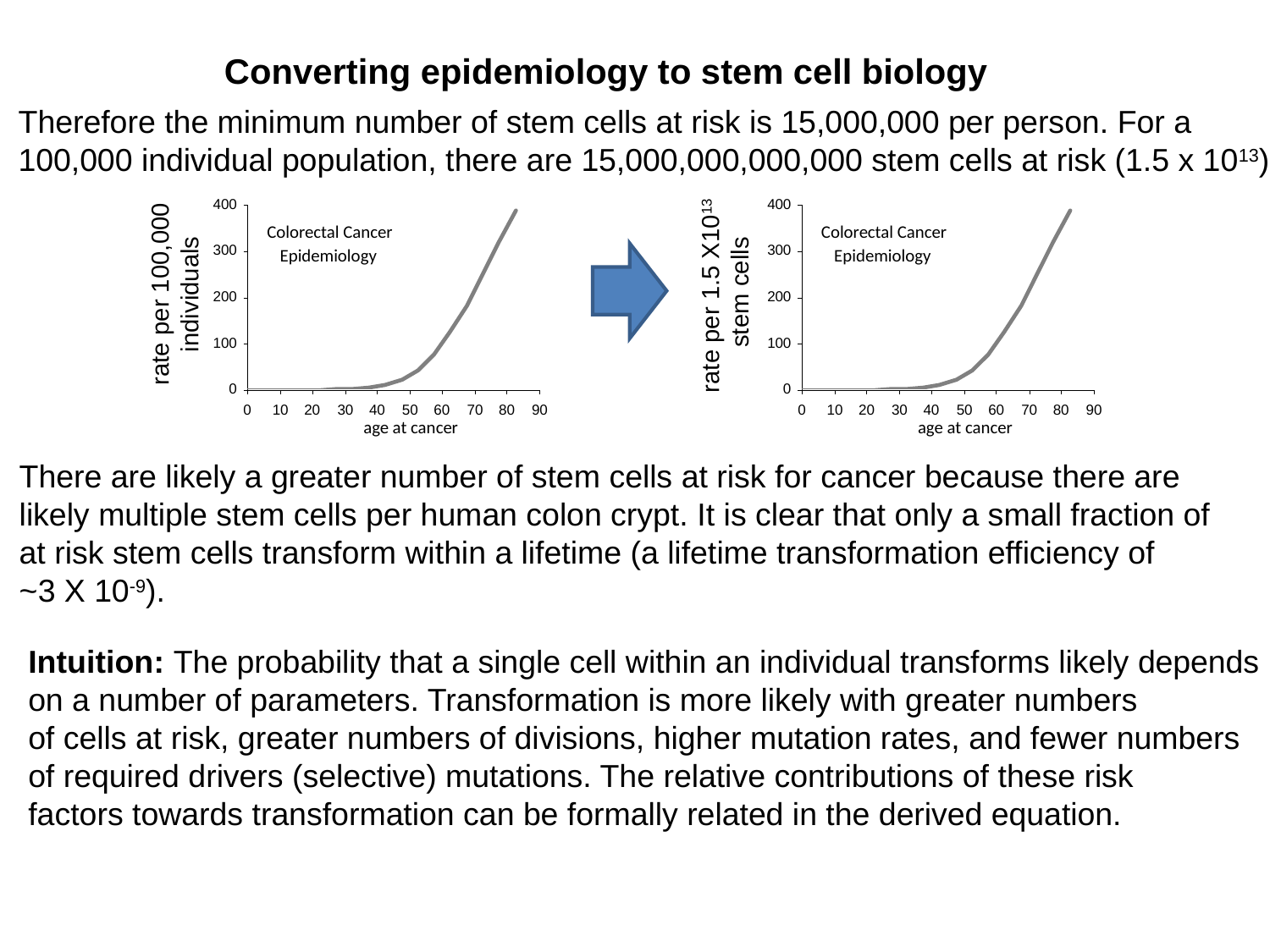

Converting epidemiology to stem cell biology
Therefore the minimum number of stem cells at risk is 15,000,000 per person. For a
100,000 individual population, there are 15,000,000,000,000 stem cells at risk (1.5 x 10^13^)
 Colorectal Cancer
 Epidemiology
 Colorectal Cancer
 Epidemiology
rate per 1.5 X1013
stem cells
rate per 100,000
individuals
age at cancer
age at cancer
There are likely a greater number of stem cells at risk for cancer because there are
likely multiple stem cells per human colon crypt. It is clear that only a small fraction of
at risk stem cells transform within a lifetime (a lifetime transformation efficiency of
~3 X 10-9).
Intuition: The probability that a single cell within an individual transforms likely depends
on a number of parameters. Transformation is more likely with greater numbers
of cells at risk, greater numbers of divisions, higher mutation rates, and fewer numbers
of required drivers (selective) mutations. The relative contributions of these risk
factors towards transformation can be formally related in the derived equation.

## Slide 3
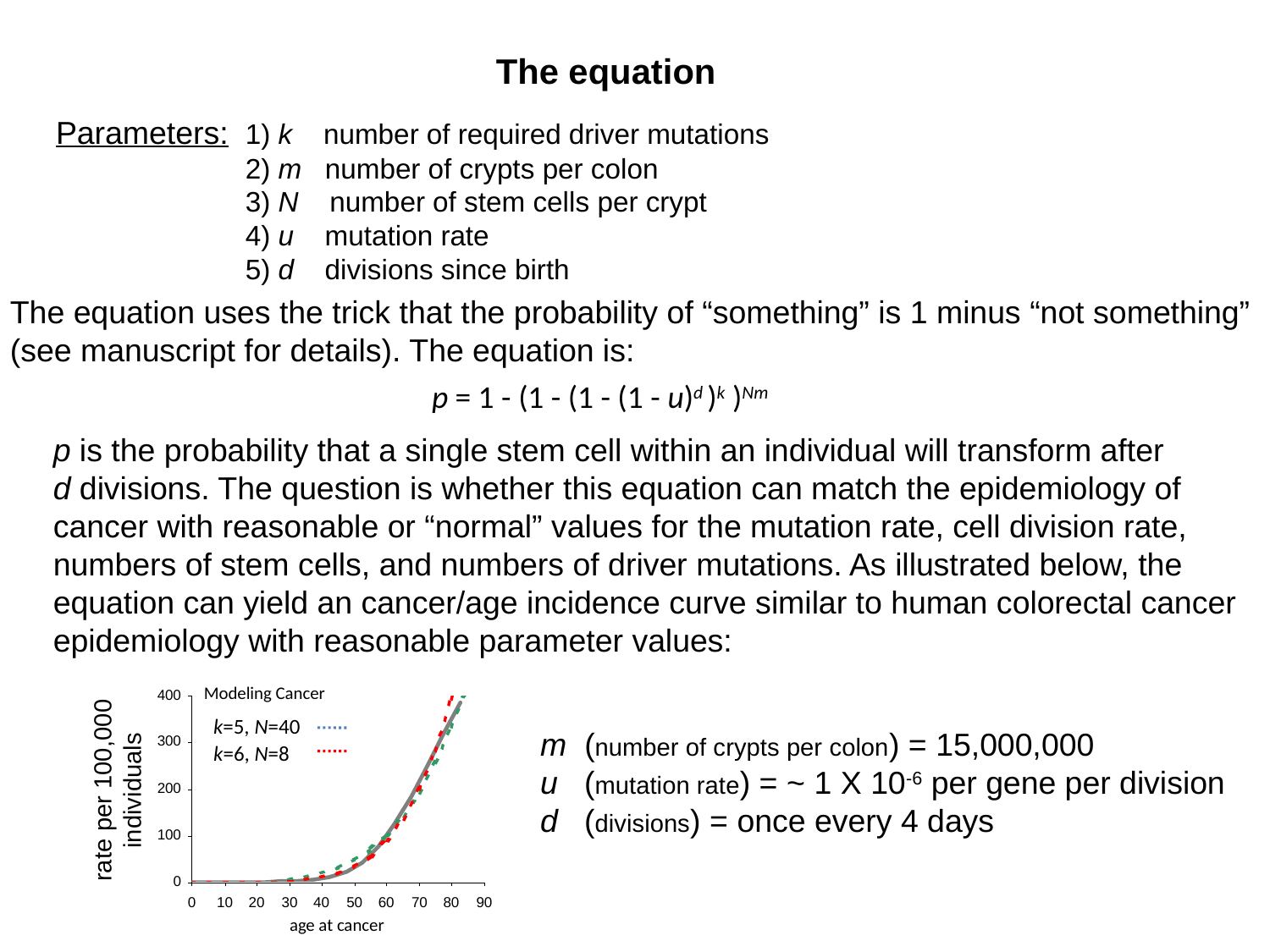

The equation
Parameters:
	1) k number of required driver mutations
	2) m number of crypts per colon
	3) N number of stem cells per crypt
	4) u mutation rate
	5) d divisions since birth
The equation uses the trick that the probability of “something” is 1 minus “not something”
(see manuscript for details). The equation is:
p = 1 - (1 - (1 - (1 - u)d )k )Nm
p is the probability that a single stem cell within an individual will transform after
d divisions. The question is whether this equation can match the epidemiology of
cancer with reasonable or “normal” values for the mutation rate, cell division rate,
numbers of stem cells, and numbers of driver mutations. As illustrated below, the
equation can yield an cancer/age incidence curve similar to human colorectal cancer
epidemiology with reasonable parameter values:
Modeling Cancer
k=5, N=40
m (number of crypts per colon) = 15,000,000
u (mutation rate) = ~ 1 X 10-6 per gene per division
d (divisions) = once every 4 days
k=6, N=8
rate per 100,000
individuals
age at cancer

## Slide 4
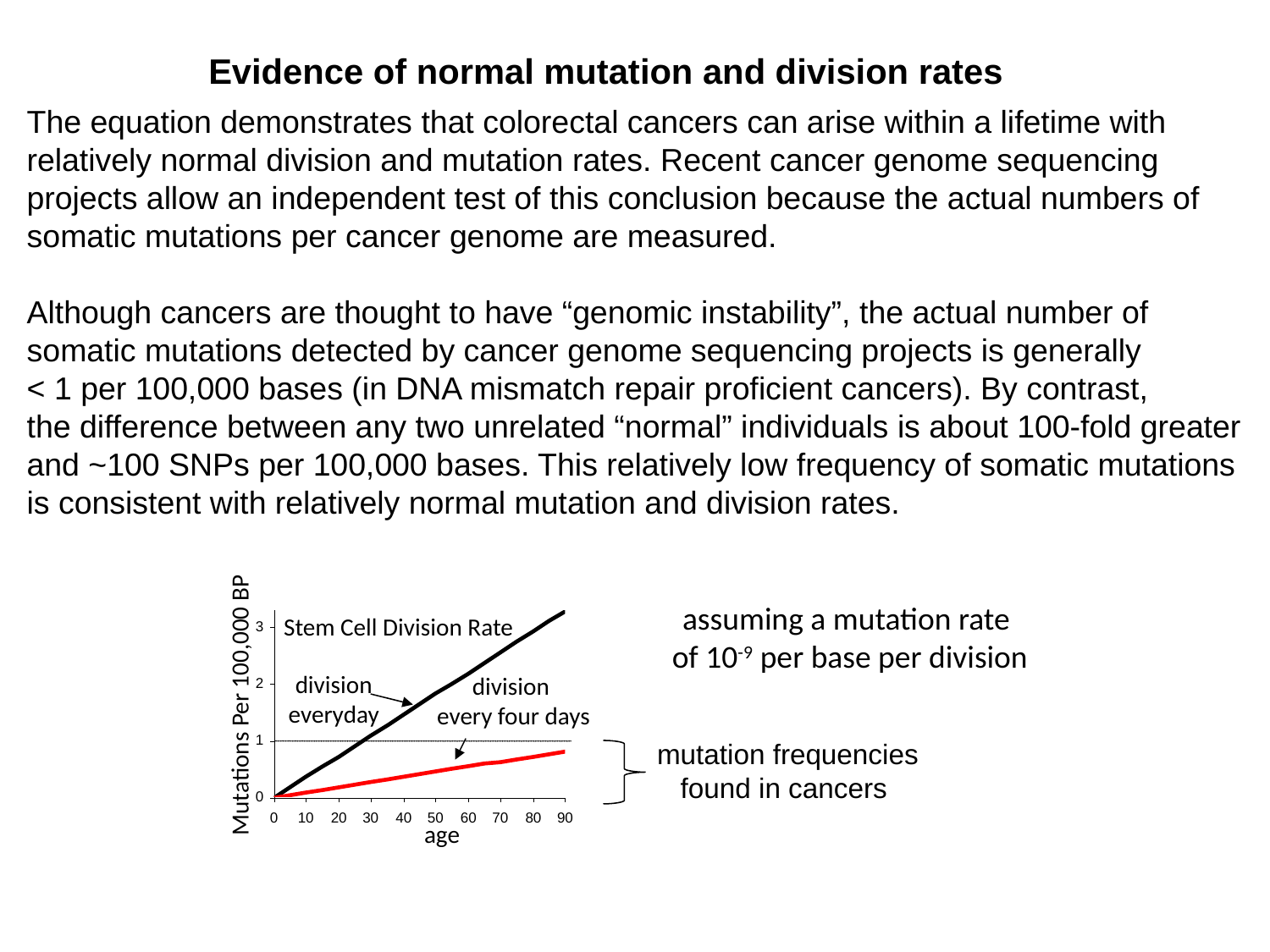

Evidence of normal mutation and division rates
The equation demonstrates that colorectal cancers can arise within a lifetime with
relatively normal division and mutation rates. Recent cancer genome sequencing
projects allow an independent test of this conclusion because the actual numbers of
somatic mutations per cancer genome are measured.
Although cancers are thought to have “genomic instability”, the actual number of
somatic mutations detected by cancer genome sequencing projects is generally
< 1 per 100,000 bases (in DNA mismatch repair proficient cancers). By contrast,
the difference between any two unrelated “normal” individuals is about 100-fold greater
and ~100 SNPs per 100,000 bases. This relatively low frequency of somatic mutations
is consistent with relatively normal mutation and division rates.
Stem Cell Division Rate
division
everyday
division
every four days
Mutations Per 100,000 BP
age
assuming a mutation rate
of 10-9 per base per division
 mutation frequencies
found in cancers

## Slide 5
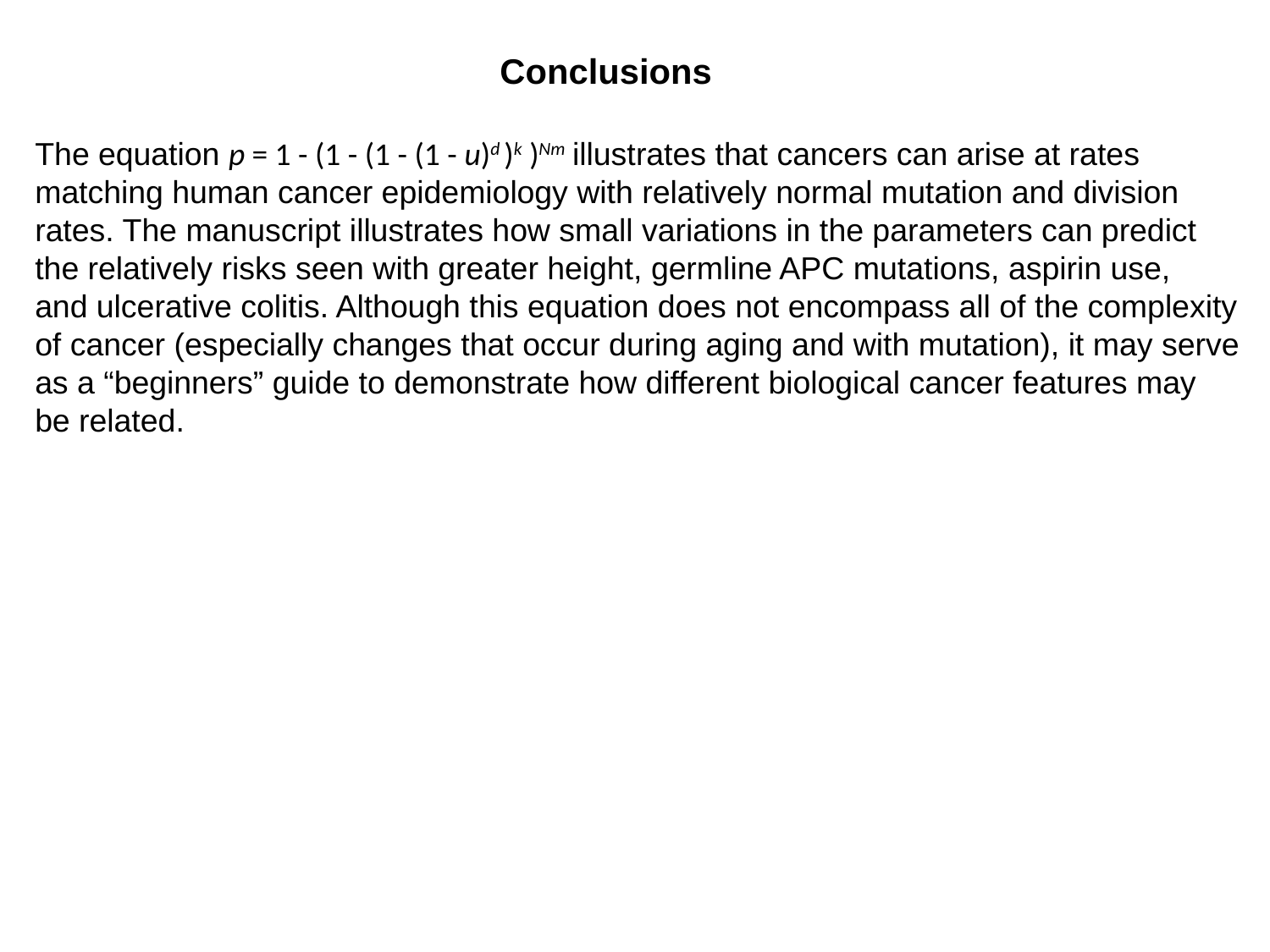

Conclusions
The equation p = 1 - (1 - (1 - (1 - u)d )k )Nm illustrates that cancers can arise at rates
matching human cancer epidemiology with relatively normal mutation and division
rates. The manuscript illustrates how small variations in the parameters can predict
the relatively risks seen with greater height, germline APC mutations, aspirin use,
and ulcerative colitis. Although this equation does not encompass all of the complexity
of cancer (especially changes that occur during aging and with mutation), it may serve
as a “beginners” guide to demonstrate how different biological cancer features may
be related.
